# Supplementary material for: Reduction in the Residues of Penthiopyrad in Processed Edible Vegetables by Various Soaking Treatments and Health Hazard Evaluation in China
Source: Foods. 2023 Feb 19;12(4):892. doi: 10.3390/foods12040892 (PMC9957162; doi:10.3390/foods12040892)
Supplement: Supplementary file 1 [file foods-12-00892-s001.zip › foods-2220770-supplementary.pdf]

Supplementary Materials

Table S1. Brief introduction of different soaking procedures.

Processing method	Processing parameters	Duration (min)
Water soaking	Water at room temperature	5, 10, 15, 20
Heated water soaking	Heated water maintained at 45 °C	5, 10, 15, 20
Ultrasound-assisted water soaking	Water at room temperature	4, 6, 8, 10
	Ultrasound frequency: 40 kHz	
Water soaking with additives	Additive: NaCl, acetic acid, LAS and ethanol	15
	Additive concentration: 0.2%, 0.5%, 1%	

Table S2. Concentrations of penthiopyrad enantiomers in soaking solution, raw and processed tomato samples in water for different durations.

Treatment	Duration (min)	Concentration (Average ± SD, *n* = 3, μg/kg)											
		**Solution**			**Pulp**			**Peel**			**Whole fruit**		
		R	***S***	***Rac***	***R***	***S***	***Rac***	***R***	***S***	***Rac***	***R***	***S***	***Rac***
Control	0	/	/	/	162±11	161±10	323±21	2261±132	2293±161	4555±294	753±29	760±27	1514±56
A	5	10±1	10±1	20±1	21±1	22±0	44±1	2064±45	2088±65	4152±109	681±10	692±7	1373±16
	10	11±0	11±0	22±0	13±1	13±1	26±2	1962±38	1994±37	3956±70	618±40	634±42	1252±82
	15	11±0	11±0	22±0	11±1	10±1	21±2	1661±32	1708±62	3370±92	553±15	574±17	1126±31
	20	12±0	13±1	25±2	4±0	4±0	8±0	1484±194	1491±183	2975±377	512±48	511±33	1023±77
B	5	22±1	23±1	45±2	69±4	63±3	132±6	1545±90	1528±60	3073±150	585±63	580±70	1165±132
	10	46±2	47±1	94±3	60±3	60±1	120±4	1298±46	1310±84	2608±129	455±88	440±92	895±180
	15	59±3	59±4	118±6	52±2	48±3	100±4	1194±21	1206±31	2400±52	328±17	320±13	648±30
	20	62±1	63±1	125±2	38±6	37±4	75±10	904±210	903±229	1807±439	283±28	279±24	562±52
C	4	8±0	8±0	17±1	118±6	118±6	236±12	1606±32	1635±24	3241±54	593±65	591±66	1184±130
	6	10±1	10±0	20±1	95±11	97±10	192±18	1250±18	1259±18	2509±36	513±21	514±23	1027±45
	8	73±0	73±1	146±1	80±5	48±2	158±6	1136±84	1159±93	2295±177	430±45	426±52	856±97
	10	88±3	88±3	176±6	9±2	9±1	18±3	881±87	900±81	1781±167	202±40	200±39	402±80

SD was standard deviation. A: Water soaking and peeling; B: Heated water soaking and peeling; C: Ultrasound-assisted soaking and peeling.

Table S3. Concentrations of penthiopyrad enantiomers in soaking solution, raw and processed tomato samples at solutions with different additives for 15 min.

Treatment	Content (%)	Concentration (Average ± SD, *n* = 3, μg/kg)											
		Solution			Pulp			Peel			Whole fruit		
		R	*S*	*Rac*	*R*	*S*	*Rac*	*R*	*S*	*Rac*	*R*	*S*	*Rac*
Control	0	/	/	/	162±11	161±10	323±21	2261±132	2293±161	4555±294	753±29	760±27	1514±56
D	0.2	80±1	76±2	157±3	5±1	5±2	10±2	1225±145	1187±144	2412±289	201±20	190±18	391±38
	0.5	83±2	82±3	165±5	4±0	4±0	7±1	976±37	940±29	1916±65	105±6	101±5	206±11
	1.0	112±9	108±7	221±16	3±0	3±0	6±0	671±9	639±11	1310±20	83±2	80±1	163±3
E	0.2	3±0	3±0	5±0	5±1	5±1	10±2	694±16	679±8	1374±24	116±8	116±9	232±17
	0.5	3±0	3±0	6±0	4±0	4±0	8±1	358±31	357±28	714±59	100±8	98±7	198±15
	1.0	3±0	3±0	6±0	3±0	3±0	7±1	134±42	126±33	260±75	58±12	58±11	115±23
F	0.2	5±0	5±0	9±0	10±1	10±0	19±1	321±22	322±18	643±40	160±16	156±18	316±34
	0.5	8±0	8±0	15±0	9±0	9±0	17±1	296±2	289±12	585±14	131±6	125±6	256±11
	1.0	12±0	12±0	23±0	8±0	8±0	16±0	267±39	254±42	520±81	116±2	114±3	230±5
G	0.2	11±0	10±1	22±1	122±11	102±9	224±20	1925±117	1860±86	3784±202	384±51	353±42	737±93
	0.5	16±0	16±0	32±1	95±14	68±16	163±30	1648±84	1575±102	3222±185	308±8	282±17	590±24
	1.0	30±1	29±0	59±1	27±1	16±2	43±3	1357±103	1294±89	2651±192	276±13	248±12	525±25

SD was standard deviation, AA was acetic acid, LAS was linear alklybezene sulfonates, and EA was ethanol. D: Water soaking with NaCl and peeling; E: Water soaking with AA and peeling; F: Water soaking with LAS and peeling; G: Water soaking with EA and peeling.

Table S4. Concentrations of penthiopyrad enantiomers in soaking solution, raw and processed cucumber samples in water for different durations.

Treatment	Duration (min)	Concentration (Average ± SD, *n* = 3, μg/kg)											
		Solution			Pulp			Peel			Whole fruit		
		R	*S*	*Rac*	*R*	*S*	*Rac*	*R*	*S*	*Rac*	*R*	*S*	*Rac*
Control	0	/	/	/	305±15	304±12	608±26	5913±250	5875±189	11788±434	2012±52	2008±54	4021±106
A	5	7±1	7±1	15±2	18±1	18±0	36±1	5284±372	5256±397	10540±769	1754±91	1812±82	3566±172
	10	15±7	15±7	29±14	15±1	15±1	29±3	4036±511	4025±428	8061±936	1595±28	1578±46	3173±71
	15	186±8	182±6	368±14	12±0	12±0	25±0	2396±260	2342±260	4738±520	783±74	804±80	1587±154
	20	234±33	231±33	465±65	12±0	12±0	23±1	671±44	626±44	1297±88	409±33	414±48	823±81
B	5	86±3	85±4	171±7	53±3	53±3	106±6	1821±70	1800±81	3622±150	118±24	122±23	241±47
	10	96±8	94±7	190±14	33±2	33±2	66±4	1676±14	1642±38	3318±50	81±3	82±3	162±6
	15	117±9	117±7	234±16	27±4	26±2	53±6	1294±237	1328±305	2622±540	66±9	67±10	133±19
	20	161±30	159±31	319±61	9±1	9±1	18±2	781±59	866±74	1738±133	43±3	44±4	87±7
C	4	62±4	61±2	123±6	139±78	125±61	264±138	4793±406	4721±391	9514±779	1680±87	1683±74	3363±161
	6	90±13	88±11	178±24	108±14	107±14	214±27	3568±122	3647±200	7214±322	1422±94	1433±76	2856±155
	8	102±2	102±3	205±5	38±25	34±18	73±43	2796±116	2751±110	5548±226	939±64	941±76	1880±140
	10	122±7	120±9	242±16	18±2	19±2	36±4	1848±222	1871±231	3718±452	798±21	788±16	1586±37

SD was standard deviation. A: Water soaking and peeling; B: Heated water soaking and peeling; C: Ultrasound-assisted soaking and peeling.

Table S5. Concentrations of penthiopyrad enantiomers in soaking solution, raw and processed cucumber samples at solutions with different additives for 15 min.

Treatment	Content (%)	Concentration (Average ± SD, n = 3, μg/kg)											
		Solution			Pulp			Peel			Whole fruit		
		R	*S*	*Rac*	*R*	*S*	*Rac*	*R*	*S*	*Rac*	*R*	*S*	*Rac*
Control	0	/	/	/	305±15	304±12	608±26	5913±250	5875±189	11788±434	2012±52	2008±54	4021±106
D	0.2	71±12	71±12	142±24	8±0	8±0	16±0	2468±198	2298±161	4766±358	823±68	784±72	1607±141
	0.5	105±10	105±11	210±20	7±1	7±1	15±1	1108±65	1090±96	2198±160	121±25	117±23	238±48
	1.0	121±4	120±4	241±8	5±0	5±0	10±0	932±19	889±34	1821±52	65±18	65±20	130±38
E	0.2	4±1	4±1	8±2	9±1	9±1	18±2	1780±38	1715±40	3495±78	42±1	42±0	84±1
	0.5	6±1	6±1	13±2	6±0	6±0	12±0	1588±110	1541±102	3129±212	38±3	37±4	75±6
	1.0	8±0	8±0	16±1	5±0	5±0	10±1	1242±52	1183±55	2426±106	31±1	30±2	60±3
F	0.2	9±0	8±0	17±1	16±1	15±1	32±2	1068±39	1036±26	2104±64	58±8	56±8	114±16
	0.5	13±0	13±0	26±1	15±0	14±1	29±1	1067±54	957±43	2023±94	47±6	42±1	88±7
	1.0	17±1	16±1	33±1	8±1	8±0	16±1	36±6	33±3	70±9	13±0	13±0	26±0
G	0.2	11±0	11±0	22±1	93±2	93±3	186±5	4421±350	4327±312	8748±662	1539±69	1542±89	3081±158
	0.5	13±1	13±1	26±2	85±7	83±8	168±15	3867±160	3728±219	7596±374	1340±154	1340±168	2679±321
	1.0	17±3	17±3	34±5	28±6	27±6	55±11	3479±221	3330±135	6809±355	957±102	928±116	1884±217

SD was standard deviation, AA was acetic acid, LAS was linear alklybezene sulfonates, and EA was ethanol. D: Water soaking with NaCl and peeling; E: Water soaking with AA and peeling; F: Water soaking with LAS and peeling; G: Water soaking with EA and peeling.

Table S6. Concentrations of penthiopyrad enantiomers in tomato sauce and cucumber juice samples after storage at room temperature (RT) and 4 °C.

Sample	Temperature	Time (d)	Concentration (Average ± SD, *n* = 3, μg/kg)			Sample	Temperature	Time (d)	Concentration (Average ± SD, *n* = 3, μg/kg)		
			R	***S***	***Rac***				***R***	***S***	***Rac***
Tomato sauce	4 °C	0	161±3	145±2	306±5	Cucumber juice	4 °C	0	63±0	64±1	128±1
		1	150±5	131±3	281±10			1	63±0	63±0	126±0
		2	129±14	123±3	252±16			2	61±1	61±1	123±2
		3	110±1	95±1	205±2			3	59±0	60±0	119±1
		5	108±0	92±1	200±1			5	57±0	59±1	116±1
		7	107±0	90±0	197±1			7	57±1	58±0	114±2
		9	105±0	89±0	194±0			9	56±0	57±1	113±2
		12	102±1	87±1	190±2			12	54±1	56±0	110±1
	RT	1	139±1	111±13	250±14		RT	1	62±0	62±0	124±1
		2	112±1	93±1	205±2			2	61±0	61±1	122±2
		3	110±2	91±0	201±2			3	58±1	58±1	116±2
		5	106±2	89±1	195±3			5	53±0	54±1	107±1
		7	105±1	88±0	193±1			7	52±0	53±1	106±2
		9	103±1	86±0	190±1			9	51±0	52±0	103±0
		12	99±2	82±2	181±4			12	50±1	50±1	100±2

SD was standard deviation.

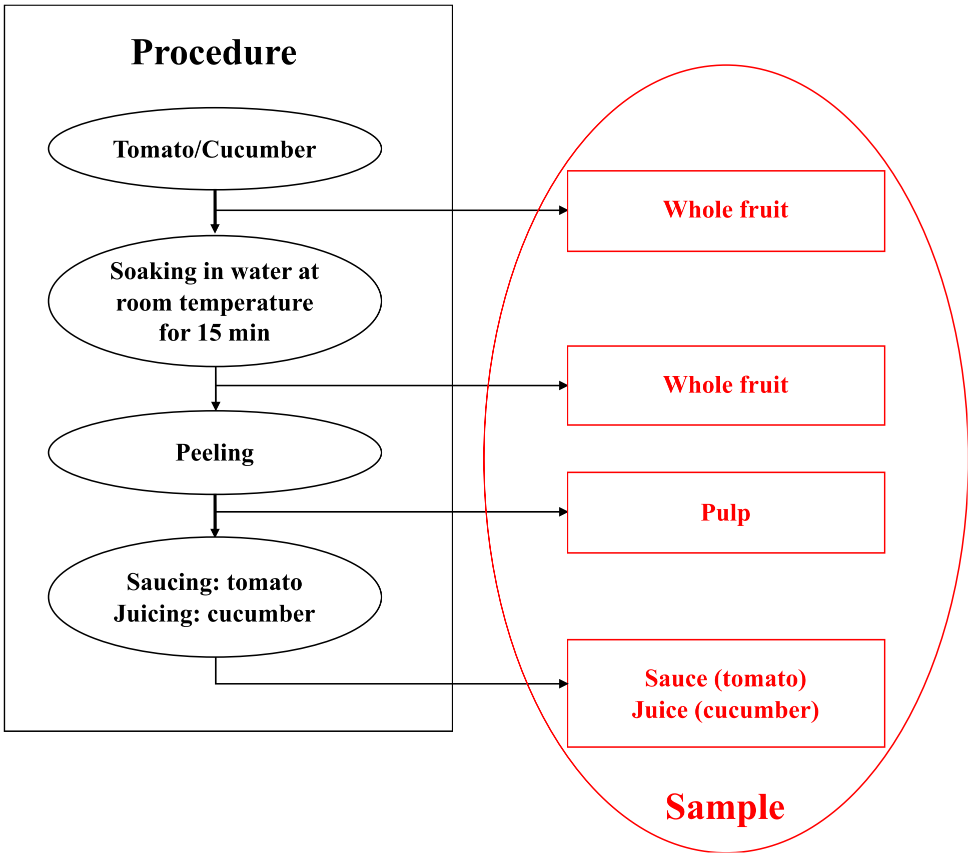

Figure S1. Procedure of saucing and juicing.

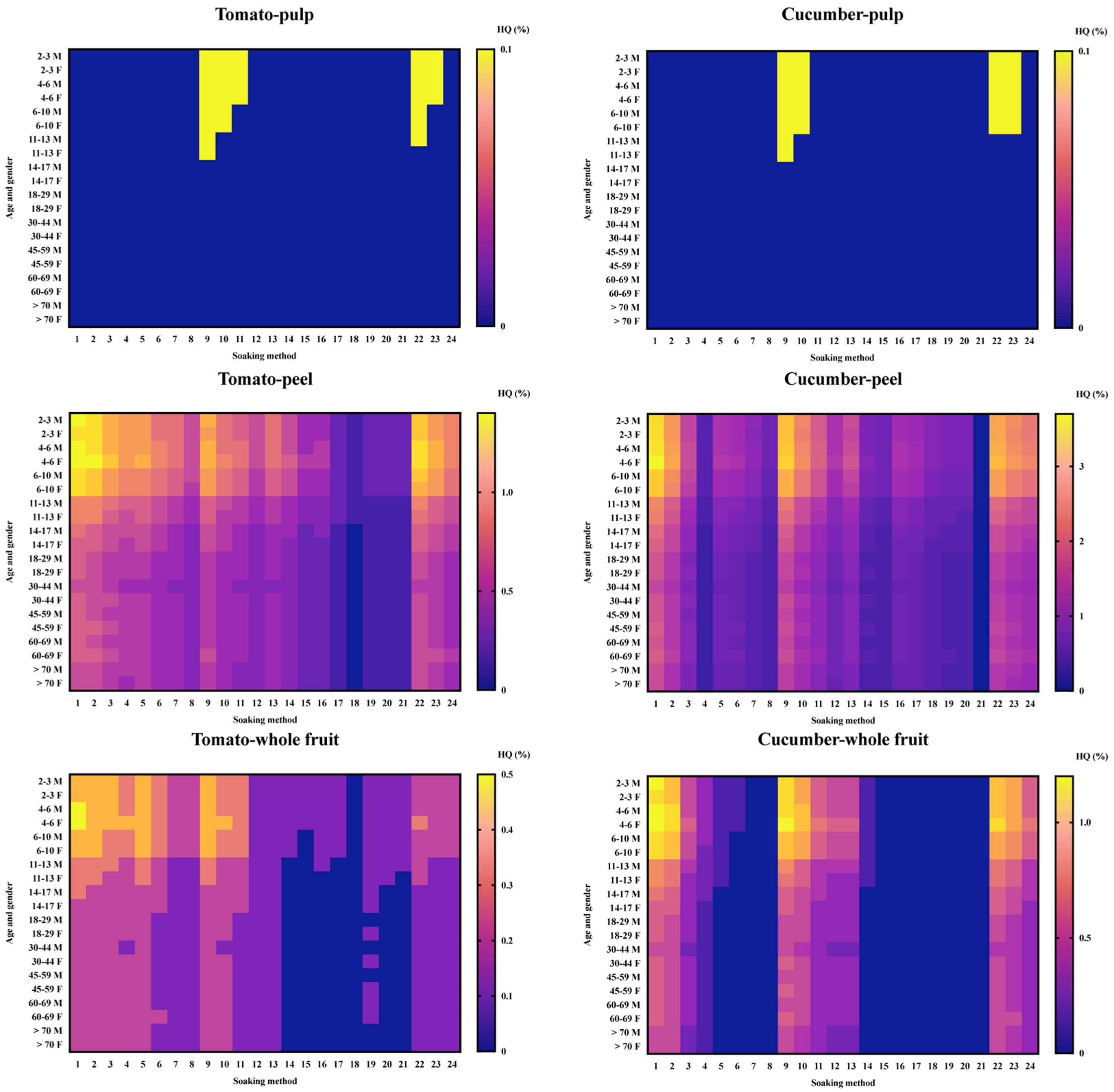

Figure S2. Heatmap of health quotient (HQ) values of penthiopyrad (racemate) in tomato and cucumber samples for different ages and genders of Chinese consumers after different soaking methods (1-4: water soaking for 5-20 min and peeling; 5-8: heated water soaking for 5-20 min and peeling; 9-12: ultrasound-assisted soaking for 4-10 min and peeling; 13-15: water soaking with NaCl (0.2%, 0.5%, 1%) and peeling; 16-18: water soaking with AA (0.2%, 0.5%, 1%) and peeling; 19-21: water soaking with LAS (0.2%, 0.5%, 1%) and peeling; 22-24: water soaking with EA (0.2%, 0.5%, 1%) and peeling).
